# Evaluation of problems encountered in daily living activities by ındividuals with colostomy: use of the Visual Analog Scale

**DOI:** 10.7717/peerj.20763

**Published:** 2026-02-11

**Authors:** Muaz Gülşen, Nursevim Aydıngülü, Sevban Arslan, Hülya Binokay

**Affiliations:** 1 Faculty of Health Sciences Surgical Nursing Department, Cukurova University, Adana, Turkey; 2 Cukurova University Faculty of Medicine, Department of Biostatistics, Cukurova University, Adana, Turkey

**Keywords:** Stoma, Colostomy, Activities of daily living, Activities of daily living model, Nursing

## Abstract

**Background:**

Colostomy is a surgical intervention that affects physical and psychological health and can cause difficulties in areas such as personal care, hygiene, nutrition, mobility, and social interactions.

**Purpose:**

To determine the severity of difficulties encountered in daily living activities by individuals with colostomy and to examine in which activities they experience the most difficulty.

**Methods:**

The research was conducted with 94 patients using a cross-sectional and descriptive design based on the Model of Living. Data were collected using the “Patient Identification Information Form” and the “Daily Living Activities Difficulty Level Assessment Form” In the first stage, the “Patient Identification Information Form” was administered to the patients, and the “Daily Living Activities Difficulty Level Assessment Form” was introduced. In the second stage, patients were contacted by telephone 15 days after discharge, and the difficulties they experienced in daily living activities were evaluated within the framework of the Daily Living Model using scores ranging from 0 to 10.

**Results:**

Patients experienced the most difficulty in areas such as excretion (8.0 ± 0.9) and anxieties about death and the future, quality of life, and meeting spiritual needs (7.0 ± 0.6), while activities with moderate difficulty were eating and drinking (4.0 ± 0.8), personal hygiene (6.0 ± 0.7), and sleep-rest (6.0 ± 0.5). The activities with the least difficulty were determined to be respiration (1.0 ± 0.4) and maintaining body temperature (2.0 ± 0.6). Patients with a higher level of education experienced less difficulty in sexual life activities. Single patients experienced less difficulty in providing a safe environment and engaging in work-leisure activities compared to married patients. Patients with temporary stomas reported experiencing less difficulty in eating and drinking activities compared to those with permanent stomas.

**Conclusion:**

The study found that the daily living activities of patients with colostomy are affected at different levels. These findings emphasize the importance of a holistic care approach addressing the physical and psychosocial needs of individuals with stomas. Future research should evaluate specific interventions aimed at improving the quality of life of these individuals.

## Introduction

Colostomy is a commonly performed surgical procedure for the treatment of colorectal cancer, inflammatory bowel diseases, and traumatic bowel injuries (Mena-Jiménez, Rodríguez-Suárez & González-de la Torre, 2024). This procedure involves creating a temporary or permanent opening in the abdominal wall to allow bowel contents to exit the body (Mena-Jiménez, Rodríguez-Suárez & González-de la Torre, 2024). Colostomy not only results in a physiological change; it also significantly affects an individual’s daily life activities, social relationships, self-care skills, and psychological adjustment process (Petersén & Carlsson, 2021; Simpson et al., 2023; Zhabagin et al., 2024). Individuals with colostomies encounter various difficulties in activities of daily living, such as mobility, personal hygiene, nutrition, stool and gas control, dressing, and social interaction (Alp, 2014; Stavropoulou et al., 2021; Wang et al., 2024; Soelling et al., 2025; Osborne et al., 2022; Abdul Khadar & Ramalingam, 2024). The persistence of these difficulties can reduce quality of life and negatively affect psychosocial adjustment (Soelling et al., 2025; Osborne et al., 2022; Abdul Khadar & Ramalingam, 2024).

International health organizations, particularly the World Council of Enterostomal Therapists (WCET) and the Australian Association of Stomal Therapy Nurses (AASTN), emphasize the importance of data based on robust measurement frameworks to develop care approaches that enhance the quality of life of individuals with colostomies (WCET, 2025; AASTN, 2025). Studies in the literature reveal important findings regarding the problems experienced by individuals with colostomies, such as pain management, hygiene, mobility limitations, fecal control, changes in dietary patterns, social isolation, and sleep problems (Alp, 2014; Stavropoulou et al., 2021; Wang et al., 2024; Soelling et al., 2025; Osborne et al., 2022; Abdul Khadar & Ramalingam, 2024; Kalayci & Duruk, 2022; Duluklu & Çelik, 2024).

Previous studies have provided important insights into the challenges individuals living with colostomy face in their daily lives. These difficulties focus particularly on daily experiences that affect physical, psychological, and social functioning, and the literature contains comprehensive descriptions on this subject (Alp, 2014; Stavropoulou et al., 2021; Wang et al., 2024; Soelling et al., 2025; Osborne et al., 2022; Abdul Khadar & Ramalingam, 2024; Kalayci & Duruk, 2022; Duluklu & Çelik, 2024). Qualitative studies have detailed the daily challenges of living with a colostomy, while quantitative studies have mostly assessed these challenges through general quality of life measures or ostomy-specific adjustment and adaptation tools (Stavropoulou et al., 2021; Osborne et al., 2022; Abdul Khadar & Ramalingam, 2024). However, a large proportion of these quantitative studies have focused on total scores or composite domains rather than addressing daily living activities individually; this may limit the identification of activity-specific difficulties (Alp, 2014; Wang et al., 2024; Soelling et al., 2025). Furthermore, the co-evaluation of different types of ostomies in many studies makes it difficult to distinguish difficulties specific to individuals with colostomies (Stavropoulou et al., 2021; Osborne et al., 2022; Abdul Khadar & Ramalingam, 2024). Therefore, the current literature highlights the need for studies that aim to quantitatively determine which daily living activities are perceived as more difficult at the individual level due to colostomy and to what extent each activity is affected, rather than a lack of evidence. It has been reported that the inability to systematically classify these needs makes it difficult to determine priority areas of care in clinical practice and to develop targeted clinical and educational interventions (Hamad, 2025; Aboma & Kaba, 2023; Yang et al., 2024).

The Visual Analog Scale (VAS) is a reliable measurement approach that allows individuals to express the severity of their difficulties numerically based on their own perceptions (Aydın, Araz & Aslan, 2011; Begum & Hossain, 2019). This scale has the potential to reveal not only the presence of colostomy-related difficulties but also the specific daily living activities they are more pronounced. Therefore, the use of VAS can enable the systematic assessment of the degree of impact on different aspects of life after colostomy and the identification of concrete priorities in care planning.

This study was conducted using an exploratory, descriptive, and cross-sectional design to identify the difficulties experienced by individuals with a colostomy in performing daily living activities and to determine which of these activities are most and least affected. The findings are expected to contribute to the development of individualized nursing care plans and patient education programs by identifying priority areas for intervention. Furthermore, the results are anticipated to enhance healthcare professionals’ awareness and support the delivery of more targeted and patient-centered post-colostomy care.

## Methods

### Type of study

This study is an exploratory, descriptive, and cross-sectional research project conducted to determine the severity of difficulties encountered in daily living activities by individuals living with colostomy. The study was reported in accordance with the Strengthening the Reporting of Observational Studies in Epidemiology (STROBE) guidelines (Babaoğlu et al., 2021).

### Location and time of study

The study was conducted with patients who underwent colostomy surgery between June 15, 2024, and March 15, 2025, at the general surgery service and intensive care unit of Çukurova University Faculty of Medicine Balcalı Hospital, Adana, Türkiye.

To increase the consistency and reliability of the data, only patients who underwent colostomy surgery were included in the study. Patients who underwent ileostomy and urostomy were not included in the study. This selection aims to minimize the differences in physiological effects and daily living activities brought about by different types of ostomies, thereby reducing the variability in the level of difficulty patients experience when performing their daily living activities.

Since colostomy, ileostomy, and urostomy surgeries are surgical procedures applied to different sections of the intestines or urinary tract, patients’ care needs, bowel movements, fluid-electrolyte balance, and stoma adaptation processes vary significantly. These differences can affect the difficulties in activities of daily living, which is the variable of the study. Including only patients with colostomy aims to increase internal validity by creating a more homogeneous sample and to ensure that the results obtained lead to more precise inferences for a specific patient group.

In addition, patients whose colostomy surgery was performed by a single surgeon were included in the study. This selection has standardized patients’ “*pre- and post-operative care processes*” by eliminating differences in surgical technique and experience. Selecting patients who were operated on by a single surgeon and who underwent only colostomy facilitated order and convenience in the data collection process, increasing the reliability of the results. Thus, it allowed for a more accurate analysis of the difficulties encountered in activities of daily living, which is the focus of the research.

#### Standard care protocol applied by the surgeon before and after surgery

Patients undergoing colostomy surgery are admitted to the clinic 3 days before the operation, and bowel preparation is initiated. From the day of admission, nutrition is regulated, oral intake is discontinued, and the patient is given an enema. Based on the examinations performed, a decision is made as to whether the surgery will be performed using an open or closed method. On the day before the operation, the surgeon marks the operation area and informs the patient and their relatives about the surgical process and aftercare.

On the 0th day post-surgery, stoma control is performed by the surgeon and ostomy nurse for the patient and their relative, and the patient is introduced to the stoma. This process aims to increase the patient’s adaptation to the stoma and encourage their participation in the treatment process. However, the education process is postponed due to intense pain in the postoperative period. On the 2nd day post-surgery, with the reduction of pain, ostomy control is performed and education is provided by the ostomy nurse. Using the demonstration method, care practices are shown, and the patient and their relative can observe the process and ask questions. On the 3rd day post-surgery, a second education is given, and the patient and their relative actively participate in the care process. Adaptor placement, bag emptying, and other care practices are taught under the guidance of the ostomy nurse; care is primarily transferred to the patient, or to their relative if the patient is unable to manage the process. Before discharge, training on daily living activities is provided, and an educational brochure is presented to ensure the retention of information. The patient and their relative’s questions are answered, and any concerns are addressed. On the 15th day post-discharge, the patient is called for a check-up at the stoma unit, and the ostomy nurse assesses the condition of the stoma and the patient’s care skills, providing additional training if necessary.

#### Determining the time of assessment of daily living activities

The follow-up period for assessing daily living activities after discharge was set to the 15th day. This timing was planned to align with the stoma nurse’s education process and the surgeon’s postoperative control schedule. The assessment schedule was determined considering the incision healing process, the clinic’s patient follow-up protocols, and the surgeons’ routine control intervals.

### Population and sample of the study

The population of the study consists of patients undergoing colostomy surgery at the General Surgery Clinic of Cukurova University Faculty of Medicine Balcalı Hospital.

The sample size was calculated using the G*Power analysis program. The sample size was determined using an estimation (precision-based) rather than a hypothesis-testing approach. The aim of the study was to estimate the mean VAS value with a predefined level of precision. The primary endpoint was the overall VAS mean for Daily Living Activities (DLA), and the target was set as a two-sided 95% confidence interval with a half-width of ±0.20 VAS units. As there were no comparable VAS data for daily living activities (DLA-VAS) available in the literature, a plausible standard deviation (SD) range of 0.80–1.00 was assumed for the 0–10 VAS scale in a clinically homogeneous cohort, and a conservative assumption of SD = 0.90 was selected (sensitivity analyses were also performed for 0.80 and 1.00). Under these assumptions, the required sample size was calculated as 78. An estimated 20% attrition rate was added (Erdoğan, Nahcıvan & Esin, 2014), and accordingly, the planned total sample size was determined as 94. The study was completed after reaching the predetermined sample size.

Patients who underwent elective colostomy surgery for the first time by the determined surgeon, who were 18 years of age or older, able to communicate verbally, did not develop postoperative complications, and were willing to participate in the research were included in the study. Among the inclusion criteria, the indications for colostomy were also considered; these included colon cancer, polyp, diverticulum, Crohn’s disease, trauma, and other causes. Patients who developed complications after surgery until the data collection process, had psychiatric disorders or treatment, or underwent ileostomy or urostomy were excluded from the scope of the research.

### Data collection tools

The “Patient Identification Information Form” and the “Daily Living Activities Difficulty Level Assessment Form” were used in the collection of data.

#### Patient identification information form

The variables in this form were determined by the researchers, supported by studies in the literature, in a manner appropriate to the aim of the research, the research questions, and the target audience (Alp, 2014; Stavropoulou et al., 2021; Kara & Aslan, 2017). In this way, it was aimed to increase the reliability and validity of the research. The form consists of items that question the sociodemographic characteristics and health status of the patients.

#### Daily living activities difficulty level assessment form (DLA-VAS)

The Level of Difficulty in the Daily Living Activities Assessment Form used in this study was adapted from a patient follow-up form previously developed based on the Roper-Logan-Tierney Model of Living and tested for validity and reliability. The original form consisted of 54 items related to patients’ daily living activities and was evaluated for content validity by an expert panel consisting of six nurses and four nursing faculty members. Following revisions made according to expert recommendations, the internal consistency (Cronbach’s alpha coefficient) of the form was determined as 0.89, and the reliability of the form was found to be high (Gülşen & Akansel, 2020). In this study, the Cronbach’s alpha coefficient was found to be 0.71.

In this study, the form was adapted to determine the severity of difficulties experienced by individuals with a colostomy while performing their daily living activities, using studies in the literature and international guidelines (Alp, 2014; Stavropoulou et al., 2021; Gülşen & Akansel, 2020; Tufekci, Akansel & Sivrikaya, 2022; Nurses Specialized in Wound & Ostomy Continence Canada-NSWOCC, 2022; UOAA, 2024; Colostomy, 2019). During the adaptation process, the item content of the form was evaluated for clinical and contextual appropriateness by three experts in ostomy and surgical nursing together with the researchers. The structure of the form is based on the 12 fundamental activities of living defined by the Roper-Logan-Tierney Model: maintaining a safe environment, communication, breathing, eating and drinking, elimination, personal hygiene, maintaining body temperature, mobility, work and recreation, sexuality, sleep and rest, and death and spiritual needs (Gülşen & Akansel, 2020; Tufekci, Akansel & Sivrikaya, 2022).

The level of difficulty for each activity of daily living is assessed using the VAS developed by Albersnagel (1998) and whose Turkish validity and reliability study was conducted by Aydın, Araz & Aslan (2011). The VAS is scored between 0 (“not affected at all”) and 10 (“so affected that the activity cannot be performed”). Score ranges are classified as 0–3 “mild,” 4–6 “moderate,” and 7–10 “severe” (Aydın, Araz & Aslan, 2011). A general evaluation is made by calculating the mean score for each activity of daily living, and a higher mean score indicates that patients experience greater difficulty in performing their daily living activities.

Furthermore, existing scales evaluating activities of daily living in the literature were examined by the research team before the form was created. As a result of the review, the Oswestry Low Back Pain Disability Index measures the effect of low back pain on daily living activities (Fairbank et al., 1980), while the Londrina Activities of Daily Living Protocol (Sant’Anna et al., 2017) assesses movements of the upper and lower extremities and the trunk. The Milliken Activities of Daily Living Scale (Akel et al., 2012) covers motor functions related to the upper extremity, while the Dementia Functional Impairment Scale (Gélinas et al., 1999) measures the level of functional independence in daily living activities of patients with dementia. Among the scales frequently used in the context of surgical diseases nursing, the Katz Index of Independence in Activities of Daily Living (Katz et al., 1963) evaluates limited basic activities such as bathing, dressing, toileting, mobility, excretion, and nutrition, while the Lawton-Brody Instrumental Activities of Daily Living Scale (Lawton & Brody, 1969) measures more complex skills such as telephone use, shopping, meal preparation, housekeeping, laundry, traveling, and medication management. As a result, it was determined that none of the existing scales comprehensively and holistically evaluated the difficulties encountered by individuals with stomas in their daily living activities, so the form was created.

#### Applicability of data collection tools

Before the study, a preliminary application was carried out to evaluate the effectiveness of the data collection process. The preliminary application was conducted with 10 patients using face-to-face interviews and telephone follow-up methods. In the first stage, after ostomy management training was given to the participants by the ostomy nurse, the “Patient Identification Information Form” was administered, and the “Daily Living Activities Difficulty Level Assessment Form” was introduced and how to fill it out was explained. In the second stage, participants were asked to evaluate their difficulty levels with the VAS scale in telephone interviews on the 15th day after discharge. During the preliminary application, the understandability of the form and the duration of the data collection process were observed, and it was determined that some minor adjustments and additions needed to be made to the items under the daily living activities headings. This stage was used to eliminate potential disruptions in the process before moving on to the main application. Patients included in the preliminary application were excluded from the scope of the study.

### Data collection

In the study, data were collected using a two-stage method consisting of face-to-face interviews and telephone follow-up.

**Figure 1:**
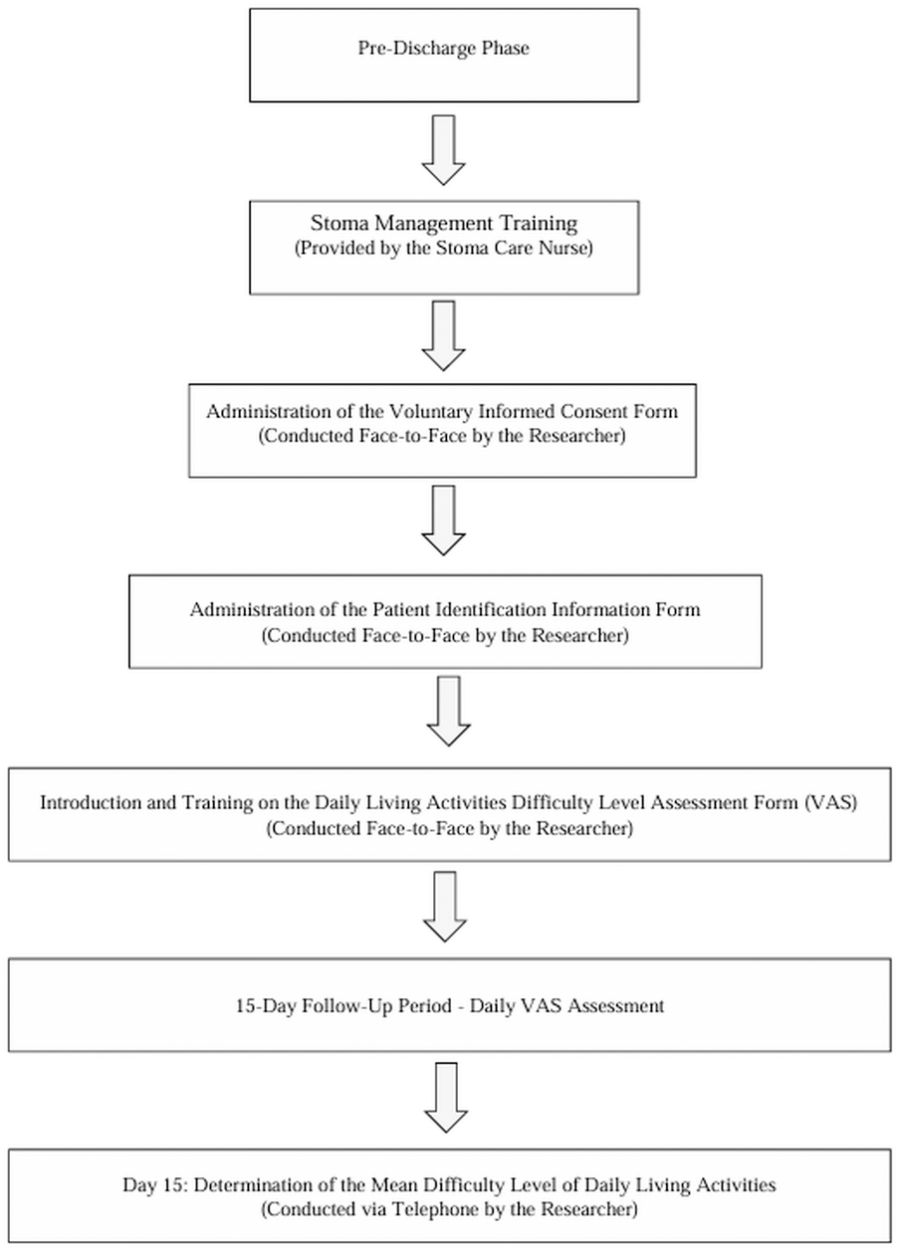
Flow diagram.

The first stage of the data collection process was carried out immediately after the patients received training on stoma management by the ostomy nurse and before discharge. Participants were given detailed information about the aim, method, and data collection process of the study by the researchers, and then patients who voluntarily agreed to participate in the study were asked to sign the “Voluntary Informed Consent Form.” Participation was conducted entirely on a voluntary basis. During the same interview, the “Daily Living Activities Difficulty Level Assessment Form” was introduced to evaluate the difficulties faced by patients in their daily living activities, and participants were explained how to fill it out (Fig. 1). Patients were asked to rate any difficulty they experienced in the daily living activities covered by the research with a score between 0 (no difficulty at all) and 10 (difficulty to the point of being unable to perform the activity). In addition, it was stated that they might not experience any difficulty in some activities throughout the 15-day follow-up period, while they might experience difficulty in other activities every day. Participants were explained that they should rate the activities they found difficult each day and calculate the average of the scores they recorded at the end of the follow-up period. Thus, for each patient, the individual difficulty level was determined by taking the average of the scores given daily for each daily living activity over the 15-day period. This method aims to reflect the general trend of the difficulties experienced by patients throughout the process, rather than one-time or accidental difficulties in a particular activity. In this way, a more reliable and comprehensive assessment of the difficulties in daily living activities after colostomy was ensured. This stage of the data collection process lasted approximately 15 min.

In the second stage, the researchers conducted telephone interviews with the patients on the 15th day after discharge, asking them to evaluate the level of difficulty they experienced in each daily living activity with a score between 0 and 10 using the VAS. During the telephone interviews, care was taken to ensure patient comfort and increase data reliability, and interviews were conducted at a time when participants were physically and psychologically suitable (Fig. 1). Patients who were not suitable were called back during the day, minimizing data loss. The second stage of the data collection process also lasted approximately 15 min, with the entire data collection process completed in a total of 30 min.

### Statistical analysis

Categorical variables were summarized as frequencies and percentages, and numerical variables as mean ± standard deviation, median, and minimum–maximum values. The Mann-Whitney U test was applied to compare non-normally distributed numerical data between two groups, and the Kruskal-Wallis test for comparisons among more than two groups. When significant differences were found, pairwise comparisons were performed using the Bonferroni-corrected Mann-Whitney U test. Statistical analyses were conducted using IBM SPSS Statistics Version 20.0 (IBM Corp., Armonk, NY, USA), with a significance level set at 0.05. Violin plots were generated using the Jamovi 2.5.3 software (The jamovi project, 2024).

### Ethical principles of the study

This study was conducted in accordance with the ethical principles stated in the World Medical Association Declaration of Helsinki. The necessary permissions were obtained from the Cukurova University Faculty of Medicine Non-Interventional Clinical Research Ethics Committee (145/73/14.06.2024) and the Balcalı Hospital Health Practice and Research Center Chief Physician’s Office. Verbal and written consent were obtained from the participants by explaining the purpose of the study.

## Results

The mean age of the patients participating in the study was 58.24 ± 11.47 years, 53.2% were male, 85.1% were married, and 24.5% were graduates of higher education. According to health data, it was determined that 35.1% of the patients had a chronic disease, 31.9% had a need for care, and 79.8% were fully independent in terms of daily living activities. In addition, colon cancer was identified as the reason for stoma creation in 40.4% of the patients (Table 1).

**Table 1:** Findings related to patients’ demographic characteristics and health status.

	**Mean ± SD**	[**Min–Max**]
	**Number (n)**	**Percentage (%)**
**Age**_**(Years)**_	58.24 ± 11.47	59 [32–79]
**Gender**		
Female	44	46.8
Male	50	53.2
**Marital status**		
Married	80	85.1
Single	14	14.9
**Education level**		
Primary education	38	40.4
Secondary education	33	35.1
Higher education	23	24.5
**Working status**		
Employed	34	36.2
Unemployed	32	34
Retired	28	29.8
**Chronic disease**		
Yes	33	35.1
No	61	64.9
**Need for Care**		
Yes	30	31.9
No	64	68.1
**Independence status in daily living activities**		
Fully	75	79.8
Partially	13	13.8
Dependent	6	6.4
**Use of assistive devices**		
Yes	33	35.1
No	61	64.9
**Reason for stoma opening**		
Colon Cancer	38	40.4
Polyp	10	10.6
Diverticulum	18	19.1
Crohn’s Disease	11	11.7
Trauma	8	8.5
Other	9	9.6
**Stoma type**		
Permanent	66	70.2
Temporary	28	29.8
**Total**	**94**	**100**

**Figure 2:**
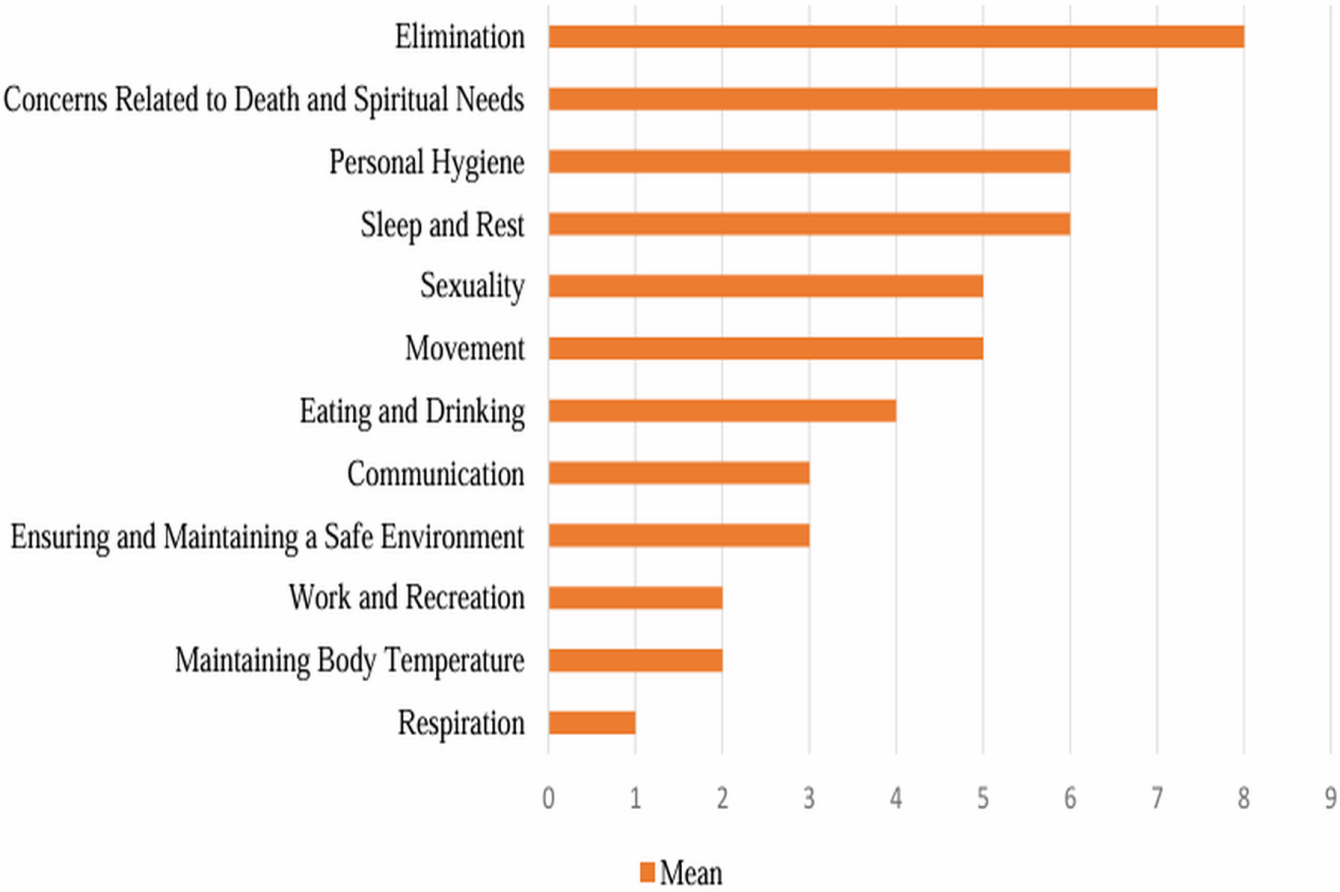
Levels of difficulty in daily living activities of patients with colostomy.

The total mean score of the difficulty level was found to be 3.0 ± 0.4 for providing and maintaining a safe environment and communication, 1.0 ± 0.4 for respiration, 4.0 ± 0.8 for eating and drinking, 8.0 ± 0.9 for excretion, 6.0 ± 0.7 for personal hygiene, 2.0 ± 0.6 for providing and maintaining body temperature, 5.0 ± 0.8 for movement, 2.0 ± 0.6 for work-leisure, 5.0 ± 0.3 for sexuality, 6.0 ± 0.5 for sleep-rest, and 7.0 ± 0.6 for anxieties about death and the future, maintaining quality of life, and spiritual needs (Table 2). It was determined that patients had more difficulty in excretion, anxieties about death and the future, maintaining quality of life, and spiritual needs activities compared to other areas, and less difficulty in work-leisure, respiration, and providing and maintaining body temperature activities (Fig. 2).

Daily living activities were affected to varying degrees in patients with colostomy (Table 3). Providing and maintaining a safe environment (83%), communication (93.6%), respiration (95.7%), providing and maintaining body temperature (97.9%), and work-leisure (95.7%) activities were slightly affected. While eating and drinking (73.4%), personal hygiene (75.5%), movement (92.6%), sexuality (100%), and sleep-rest (100%) activities were moderately affected, excretion (86.2%) and anxieties about death and the future, maintaining quality of life, and spiritual needs (83%) were the most affected areas, with patients experiencing severe difficulty in these activities (Table 3).

**Table 2:** Average difficulty level of patients in activities of daily living.

	**Mean ± SD**	**Median** [**Min–Max**]
**1. Difficulty level in providing and maintaining a safe environment**		
Pain management	2.0 ± 0.8	2 [0–3]
Medication management	2.0 ± 0.6	2 [1–5]
Bleeding control	5.0 ± 1.4	5 [1–8]
Supply procurement	1.0 ± 0.5	1 [0–1]
Skin integrity	5.0 ± 1.8	5 [0–8]
Infection management	2.0 ± 0.6	2 [1–5]
Emergency management	2.0 ± 0.8	2 [0–3]
***Total Average of Difficulty Level***	3.0 ± 0.4	3 [2–4]
**2. Level of difficulty in communication-related activities**		
Verbal communication	2.0 ± 0.5	2 [0–3]
Contact by phone	2.0 ± 0.5	2 [0–4]
Self-expression	5.0 ± 0.7	5 [3–7]
***Total Average of Difficulty Level***	3.0 ± 0.4	3 [1–4]
**3. Level of difficulty in respiratory activities**		
Breathing	1.0 ± 0.5	1 [0–3]
Coughing and sneezing	2.0 ± 0.8	2 [0–3]
***Total Average of Difficulty Level***	1.0 ± 0.4	2 [0–2]
**4. Level of difficulty in activities related to eating and drinking**		
Dietary pattern	5.0 ± 1.4	5 [1–7]
Fluid consumption	4.0 ± 1.3	5 [1–8]
Vitamin intake	3.0 ± 0.9	3 [0–5]
***Total Average of Difficulty Level***	4.0 ± 0.8	4 [2–6]
**5. Level of difficulty in activities related to excretion**		
Diarrhea and constipation management	8.0 ± 1.6	8 [3–9]
Gas management	8.0 ± 1.3	8 [4–9]
Excretion process	8.0 ± 1.4	8 [4–9]
***Total Average of Difficulty Level***	8.0 ± 0.9	8 [5–9]
**6. Level of difficulty in activities related to personal hygiene**		
Dressing up	6.0 ± 1.2	6 [2–8]
Bathing	6.0 ± 1.2	6 [2–8]
Peristomal care management	6.0 ± 1.2	6 [3–8]
***Total Average of Difficulty Level***	6.0 ± 0.7	6 [4–8]
**7. Level of difficulty in activities related to maintaining body temperature**		
Change of clothes	3.0 ± 1.0	3 [0–5]
Temperature change	1.0 ± 0.6	1 [0–2]
***Total Average of Difficulty Level***	2.0 ± 0.6	2 [0–3]
**8. Level of difficulty in movement-related activities**		
Climbing up and down stairs	5.0 ± 1.5	5 [2–8]
Walking and exercising	5.0 ± 1.2	5 [2–8]
Driving	5.0 ± 1.4	5 [1–8]
***Total Average of Difficulty Level***	5.0 ± 0.8	5 [3–7]
**9. Level of difficulty in activities related to work and leisure**		
TV-book	1.0 ± 0.9	2 [0–2]
Social activity	2.0 ± 0.7	2 [0–3]
Work management such as professional, social and household chores	2.0 ± 0.8	2 [0–3]
***Total Average of Difficulty Level***	2.0 ± 0.6	2 [0–2]
**10. Level of difficulty in sexual activities**		
Sexual compatibility	5.0 ± 0.6	5 [4–6]
Proximity	5.0 ± 0.5	5 [4–6]
Body perception management	5.0 ± 0.5	5 [4–6]
***Total Average of Difficulty Level***	5.0 ± 0.3	5 [4–6]
**11. Level of difficulty in activities related to sleep and rest**		
Falling asleep and resting	6.0 ± 0.6	6 [3–6]
Providing sleep positioning	5.0 ± 0.5	5 [4–6]
***Total Average of Difficulty Level***	6.0 ± 0.5	6 [4–6]
**12. Level of difficulty in activities related to death-future concerns and spiritual needs**		
Future anxiety management	8.0 ± 1.2	8 [3–9]
Achieving and maintaining quality of life	8.0 ± 1.0	8 [4–9]
Providing and sustaining spiritual needs such as worship	5.0 ± 0.6	5 [3–6]
***Total average of difficulty level***	7.0 ± 0.6	7 [5–8]

**Notes.**

VAS scores range from 0 (not affected at all) to 10 (severely affected), with higher scores indicating greater difficulty in performing activities of daily living.

When Table 4 is examined, there was no statistically significant difference between the average daily living activity scores when stratified by gender, age group, presence of chronic disease, need for care, independence status, and use of assistive devices (*p* > 0.05). However, the average score for activities related to sexual life was found to be lower in patients with a higher level of education (*p* < 0.05). In addition, daily living activity scores for providing and maintaining a safe environment and for work-leisure were found to be lower in single patients compared to married patients (*p* < 0.05). The daily living activity score related to eating and drinking was found to be lower in patients with temporary stomas compared to those with permanent stomas (*p* < 0.05).

**Figure 3:**
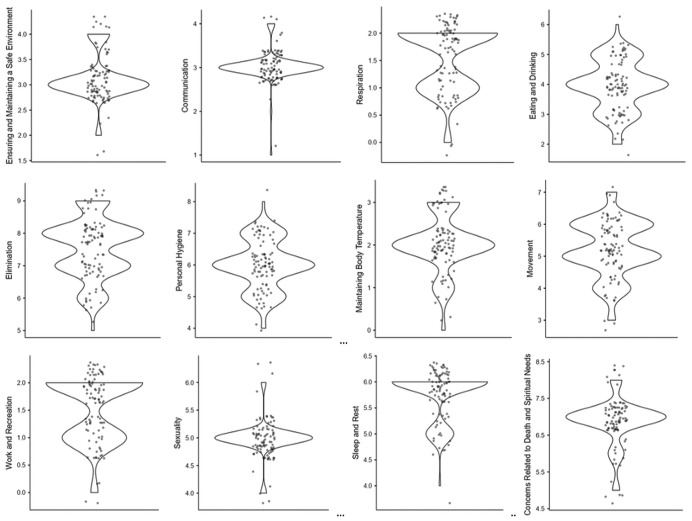
Distribution of the areas with the highest levels of difficulty in activities of daily living based on visual analog scale scores in patients with colostomies.

When Fig. 3 is examined, it is observed that the difficulty scores for activities related to providing and maintaining a safe environment and communication are concentrated around an average of 3, while the difficulty scores for activities related to eating and drinking are concentrated around 4. While the difficulty scores for activities related to excretion are around an average of 7 and 8, the difficulty scores for activities related to personal hygiene are concentrated around 6. The difficulty score for activities related to sexuality is around 5, and the difficulty score for activities related to sleep-rest is concentrated around 6. It was observed that the level of difficulty for activities related to anxieties about death and the future, maintaining quality of life, and spiritual needs is around 7 (Fig. 3).

**Table 3:** Patients’ level of difficulty in activities of daily living.

	**Number (n)**	**Percentage (%)**	
**1. Level of difficulty in providing and maintaining a safe environment**			
Mildly affected	78	83	
Moderately affected	16	17	
**2. Level of difficulty in communication-related activities**		
Mildly affected	88	93.6	
Moderately affected	6	6.4	
**3. Level of difficulty in respiratory activities**		
Not affected at all	4	4.3	
Mildly affected	90	95.7	
**4. Level of difficulty in activities related to eating and drinking**		
Mildly affected	25	26.6	
Moderately affected	69	73.4	
**5. Level of difficulty in activities related to excretion**		
Moderately affected	13	13.8	
Severely affected	81	86.2	
**6. Level of difficulty in activities related to personal hygiene**		
Moderately affected	71	75.5	
Severely affected	23	24.5	
**7. Level of difficulty in activities related to maintaining body temperature**		
Not affected at all	2	2.1	
Mildly affected	92	97.9	
**8. Level of difficulty in movement-related activities**		
Mildly affected	3	3.2	
Moderately affected	87	92.6	
Severely affected	4	4.3	
**9. Level of difficulty in activities related to work and leisure**		
Not affected at all	4	4.3	
Mildly affected	90	95.7	
**10. Level of difficulty in sexual activities**		
Moderately affected	94	100	
**11. Level of difficulty in activities related to sleep and rest**		
Moderately affected	94	100	
**12. Level of difficulty in activities related to death-future concerns and spiritual needs**		
Moderately affected	16	17	
Severely affected	78	83	

**Table 4:** Comparison of activities of daily living in colostomy patients according to demographic and health characteristics.

	**Mean ± sd, Median** [**Min–Max**]											
	**Providing and maintaining a safe environment**	**Communication**	**Respiration**	**Eating** ** and** ** drinking**	**Excretion**	**Personal hygiene**	**Providing and maintaining body temperature**	**Movement**	**Work** ** and** **entertainment**	**Sexuality**	**Sleep** **and** ** rest**	**Death-future anxieties** ** and** ** spiritual needs**
**Gender**												
Male	3.0 ± 0.5 3.0 [2–4]	5.0 ± 0.9 5.0 [3–7]	2.0 ± 0.5 2.0 [0–2]	4.0 ± 0.9 4.0 [2–5]	8.0 ± 0.9 8.0 [6–9]	6.0 ± 0.7 6.0 [4–7]	2.0 ± 0.7 2.0 [0–3]	5.0 ± 0.9 5.0 [3–7]	5.0 ± 0.3 5.0 [4–6]	5.0 ± 0.3 5.0 [4–6]	6.0 ± 0.4 6.0 [5–6]	7.0 ± 0.6 7.0 [5–8]
Female	3.0 ± 0.4 3.0 [3–4]	5.0 ± 0.8 5.0 [4–7]	2.0 ± 0.6 2.0 [0–2]	4.0 ± 0.8 4.0 [2–6]	8.0 ± 0.9 8.0 [5–9]	6.0 ± 0.8 6.0 [4–8]	2.0 ± 0.6 2.0 [0–3]	5.0 ± 0.8 5.0 [4–7]	5.0 ± 0.3 5.0 [4–6]	5.0 ± 0.3 5.0 [4–6]	6.0 ± 0.5 6.0 [4–6]	7.0 ± 0.7 7.0 [5–8]
*p*	0.134	0.536	0.627	0.422	0.628	0.738	0.492	0.923	0.601	0.327	0.691	0.344
**Age groups**												
18–44	3.0 ± 0.5 3.0 [2–4]	5.0 ± 0.9 5.0 [3–6]	2.0 ± 0.5 1.0 [1–2]	4.0 ± 1.0 4.0 [2–5]	7.0 ± 1.0 7.0 [5–9]	6.0 ± 0.8 6.0 [5–7]	2.0 ± 0.5 2.0 [1–3]	5.0 ± 0.9 5.0 [3–6]	5.0 ± 0.4 5.0 [4–6]	5.0 ± 0.4 5.0 [4–6]	6.0 ± 0.5 6.0 [5–6]	7.0 ± 0.8 7.0 [5–8]
45-64	3.0 ± 0.4 3.0 [2–4]	5.0 ± 1.0 5.0 [3–7]	2.0 ± 0.6 2.0 [0–2]	4.0 ± 0.8 4.0 [2–5]	8.0 ± 0.9 8.0 [6–9]	6.0 ± 0.8 6.0 [4–8]	2.0 ± 0.7 2.0 [0–3]	5.0 ± 1.0 5.0 [3–7]	5.0 ± 0.3 5.0 [4–6]	5.0 ± 0.3 5.0 [4–6]	6.0 ± 0.4 6.0 [4–6]	7.0 ± 0.5 7.0 [5–8]
65+	3.0 ± 0.4 3.0 [2–4]	5.0 ± 0.7 5.0 [4–6]	2.0 ± 0.7 2.0 [0–2]	4.0 ± 0.7 4.0 [3–6]	7.0 ± 0.8 8.0 [6–9]	6.0 ± 0.6 6.0 [5–7]	2.0 ± 0.6 2.0 [1–3]	5.0 ± 0.7 5.0 [4–69	5.0 ± 0.2 5.0 [5–6]	5.0 ± 0.2 5.0 [5–6]	6.0 ± 0.5 6.0 [5–6]	7.0 ± 0.7 7.0 [5–8]
*p*	0.499	0.671	0.722	0.553	0.431	0.409	0.904	0.990	0.131	0.582	0.139	0.391
**Educational level**												
Primary education	3.0 ± 0.5 3.0 [2–4]	3.0 ± 0.3 3.0 [3–4]	2.0 ± 0.6 2.0 [0–2]	4.0 ± 0.7 4.0 [3–6]	8.0 ± 0.8 8.0 [6–9]	6.0 ± 0.7 6.0 [5–8]	2.0 ± 0.7 2.0 [0–3]	5.0 ± 0.8 5.0 [4–7]	2.0 ± 0.6 2.0 [0–2]	5.0 ± 0.3 5.0 [4–6]	6.0 ± 0.5 6.0 [4–6]	7.0 ± 0.6 7.0 [5–8]
Secondary Education	3.0 ± 0.4 3.0 [3–4]	3.0 ± 0.2 3.0 [2–3]	2.0 ± 0.6 2.0 [0–2]	4.0 ± 0.9 4.0 [2–5]	8.0 ± 0.9 8.0 [6–9]	6.0 ± 0.8 6.0 [4–7]	2.0 ± 0.6 2.0 [1–3]	5.0 ± 1.0 5.0 [3–7]	2.0 ± 0.5 2.0 [0–2]	5.1 ± 0.3 5.0 [5–6]	6.0 ± 0.4 6.0 [5–6]	7.0 ± 0.6 7.0 [5–8]
Higher Education	3.0 ± 0.4 3.0 [2–4]	3.0 ± 0.6 3.0 [1–4]	2.0 ± 0.5 2.0 [1–2]	4.0 ± 0.9 4.0 [2–5]	7.0 ± 1.1 8.0 [5–9]	6.0 ± 0.9 6.0 [4–7]	2.0 ± 0.6 2.0 [1–3]	5.0 ± 0.9 5.0 [3–6]	2.0 ± 0.6 2.0 [0–2]	4.9 ± 0.3 5.0 [4–5]	6.0 ± 0.4 6.0 [5–6]	7.0 ± 0.7 7.0 [5–8]
*p*	0.294	0.199	0.397	0.258	0.757	0.091	0.418	0.792	0.417	0.030	0.662	0.782
**Marital status**												
Married	3.2 ± 0.4 3.0 [2–4]	3.0 ± 0.3 3.0 [2–4]	2.0 ± 0.6 2.0 [0–2]	4.0 ± 0.8 4.0 [2–6]	8.0 ± 0.9 8.0 [6–9]	6.0 ± 0.8 6.0 [4–8]	2.0 ± 0.6 2.0 [0–3]	5.0 ± 0.9 5.0 [3–7]	1.6 ± 0.5 2.0 [0–2]	5.0 ± 0.3 5.0 [4–6]	6.0 ± 0.5 6.0 [4–6]	7.0 ± 0.6 7.0 [5–8]
Single	2.9 ± 0.5 3.0 [2–4]	3.0 ± 0.6 3.0 [1–4]	2.0 ± 0.7 2.0 [0–2]	4.0 ± 0.9 3.5 [2–5]	8.0 ± 1.1 7.5 [5–9]	6.0 ± 0.8 6.0 [5–7]	2.0 ± 0.7 2.0 [0–3]	5.0 ± 0.8 5.0 [3–6]	1.2 ± 0.7 1.0 [0–2]	5.0 ± 0.4 5.0 [4–5]	6.0 ± 0.4 6.0 [5–6]	7.0 ± 0.8 7.0 [5–8]
*p*	0.020	0.561	0.839	0.054	0.857	0.408	0.189	0.595	0.044	0.084	0.378	0.950
**Working status**												
Employed	3.0 ± 0.5 3.0 [2–4]	3.0 ± 0.5 3.0 [1–4]	2.0 ± 0.6 2.0 [0–2]	4.0 ± 0.9 4.0 [2–5]	8.0 ± 1.0 8.0 [5–9]	6.0 ± 0.9 6.0 [4–7]	2.0 ± 0.6 2.0 [1–3]	5.0 ± 1.0 5.0 [3–7]	2.0 ± 0.6 2.0 [0–2]	5.0 ± 0.4 5.0 [4–6]	6.0 ± 0.4 6.0 [5–6]	7.0 ± 0.5 7.0 [5–8]
Unemployed	3.0 ± 0.5 3.0 [2–4]	3.0 ± 0.2 3.0 [3–4]	2.0 ± 0.6 2.0 [0–2]	4.0 ± 0.7 4.0 [3–6]	8.0 ± 0.9 8.0 [6–9]	6.0 ± 0.7 6.0 [5–8]	2.0 ± 0.6 2.0 [1–3]	5.0 ± 0.8 5.0 [4–7]	2.0 ± 0.6 2.0 [0–2]	5.0 ± 0.3 5.0 [4–6]	6.0 ± 0.4 6 [5–6]	7.0 ± 0.6 7.0 [5–8]
Retired	3.0 ± 0.5 3.0 [2–9]	3.0 ± 0.3 3.0 [3–4]	2.0 ± 0.6 2.0 [0–2]	4.0 ± 0.8 4.0 [2–5]	7.0 ± 0.9 8.0 [6–9]	6.0 ± 0.6 6.0 [5–7]	2.0 ± 0.8 2.0 [0–3]	5.0 ± 0.8 6.0 [3–6]	2.0 ± 0.5 2.0 [1–2]	5.0 ± 0.2 5.0 [5–6]	6.0 ± 0.5 6 [4–6]	7.0 ± 0.7 7.0 [5–8]
*p*	0.581	0.577	0.973	0.929	0.604	0.119	0.981	0.161	0.859	0.638	0.415	0.037
**Chronic disease**												
Yes	3.0 ± 0.4 3.0 [2–4]	3.0 ± 0.2 3.0 [3–4]	2.0 ± 0.6 2.0 [0–2]	4.0 ± 0.6 4.0 [3–5]	8.0 ± 0.9 7.0 [6–9]	6.0 ± 0.7 6.0 [5–7]	2.0 ± 0.7 2.0 [0–3]	5.0 ± 0.8 5.0 [3–6]	2.0 ± 0.6 2.0 [0–2]	5.0 ± 0.3 5.0 [4–6]	6.0 ± 0.5 6.0 [5–6]	7.0 ± 0.6 7.0 [5–8]
No	3.0 ± 0.5 3.0 [2–4]	3.0 ± 0.4 3.0 [1–4]	2.0 ± 0.6 2.0 [0–2]	4.0 ± 0.9 4.0 [2–6]	8.0 ± 1.0 8.0 [5–9]	6.0 ± 0.8 6.0 [4–8]	2.0 ± 0.6 2.0 [0–3]	5.0 ± 0.9 5.0 [3–7]	2.0 ± 0.6 2.0 [0–2]	5.0 ± 0.3 5.0 [4–6]	6.0 ± 0.5 6.0 [4–6]	7.0 ± 0.6 7.0 [5–8]
*p*	0.894	0.670	0.518	0.929	0.451	0.349	0.578	0.330	0.927	0.653	0.549	0.930
**Need for Care**												
Yes	3.2 ± 0.5 3.0 [2–4]	3.1 ± 0.3 3.0 [3–4]	1.5 ± 0.6 2.0 [0–2]	4.1 ± 0.7 4.0 [3–6]	7.4 ± 0.8 7.5 [6–9]	6.2 ± 0.8 6.0 [5–8]	2.0 ± 0.6 2.0 [1–3]	5.0 ± 0.7 5.0 [4–6]	1.6 ± 0.6 2.0 [0–2]	5.0 ± 0.2 5.0 [5–6]	5.7 ± 0.5 6.0 [4–6]	6.8 ± 0.7 7.0 [5–8]
No	3.1 ± 0.4 3.0 [2–4]	3.0 ± 0.4 3.0 [1–4]	1.6 ± 0.6 2.0 [0–2]	3.9 ± 0.9 4.0 [2–5]	7.6 ± 1.0 8.0 [5–9]	5.9 ± 0.8 6.0 [4–7]	2.0 ± 0.7 2.0 [0–3]	5.0 ± 1.0 5.0 [3–7]	1.5 ± 0.6 2.0 [0–2]	5.0 ± 0.4 5.0 [4–6]	5.8 ± 0.4 6.0 [5–6]	6.9 ± 0.6 7.0 [5–8]
*p*	0.561	0.592	0.715	0.565	0.194	0.095	0.669	0.776	0.544	0.344	0.558	0.464
**Use of Assistive Devices**												
Yes	3.2 ± 0.4 3.0 [2–4]	3.1 ± 0.2 3.0 [3–4]	1.5 ± 0.6 2.0 [0–2]	4.2 ± 0.7 4.0 [3–6]	7.4 ± 0.8 7.0 [6–9]	6.2 ± 0.8 6.0 [5–8]	2.0 ± 0.6 2.0 [1–3]	5.0 ± 0.7 5.0 [4–7]	1.6 ± 0.6 2.0 [0–2]	5.0 ± 0.2 5.0 [5–6]	5.7 ± 0.5 6.0 [4–6]	6.8 ± 0.7 7.0 [5–8]
No	3.1 ± 0.5 3.0 [2–4]	3.0 ± 0.4 3.0 [1–4]	1.6 ± 0.6 2.0 [0–2]	3.9 ± 0.9 4.0 [2–5]	7.6 ± 1.0 8.0 [5–9]	5.9 ± 0.8 6.0 [4–7]	2.0 ± 0.7 2.0 [0–3]	5.0 ± 1.0 5.0 [3–7]	1.5 ± 0.6 2.0 [0–2]	5.0 ± 0.4 5.0 [4–6]	5.8 ± 0.4 6.0 [5–6]	6.9 ± 0.6 7.0 [5–8]
*p*	0.714	0.670	0.518	0.149	0.140	0.183	0.636	0.807	0.466	0.344	0.829	0.312
**Independence status in daily living activities**											
Fully	3.2 ± 0.5 3.0 [2–4]	3.0 ± 0.3 3.0 [1–4]	1.5 ± 0.6 2.0 [0–2]	3.9 ± 0.9 4.0 [2–5]	7.6 ± 0.9 8.0 [5–9]	6.0 ± 0.8 6.0 [4–8]	2.0 ± 0.7 2.0 [0–3]	5.0 ± 0.9 5.0 [3–7]	1.5 ± 0.6 2.0 [0–2]	5.0 ± 0.3 5.0 [4–6]	5.8 ± 0.5 6.0 [4–6]	6.9 ± 0.6 7.0 [5–8]
Partially	3.1 ± 0.5 3.0 [2–4]	3.2 ± 0.4 3.0 [3–4]	1.8 ± 0.4 2.0 [1–2]	4.2 ± 0.8 4.0 [3–6]	7.5 ± 0.9 8.0 [6–9]	6.1 ± 0.8 6.0 [5–7]	2.1 ± 0.5 2.0 [1–3]	5.0 ± 0.7 6.0 [4–6]	1.7 ± 0.6 2.0 [0–2]	5.0 ± 0.3 5.0 [5–6]	5.9 ± 0.4 6.0 [5–6]	6.8 ± 0.9 7.0 [5–8]
Dependent	3.0 ± 0.0 3.0 [3–3]	3.0 ± 0.0 3.0 [3–3]	1.3 ± 0.8 1.5 [0–2]	4.0 ± 0.6 4.0 [3–5]	7.3 ± 0.8 7.5 [6–8]	6.3 ± 0.5 6.0 [6–7]	2.3 ± 0.8 2.5 [1–3]	5.0 ± 0.6 5.0 [4–6]	1.7 ± 0.5 2.0 [1–2]	5.0 ± 0.0 5.0 [5–5]	5.5 ± 0.5 5.5 [5–6]	6.8 ± 0.4 7.0 [6–7]
*p*	0.650	0.319	0.284	0.806	0.775	0.426	0.422	0.396	0.331	0.538	0.254	0.936
**Stoma Type**												
Permanent	3.1 ± 0.4 3.0 [2–4]	3.0 ± 0.4 3.0 [1–4]	1.6 ± 0.6 2.0 [0–2]	4.1 ± 0.7 4.0 [2–6]	7.6 ± 0.8 8.0 [6–9]	6.0 ± 0.7 6.0 [4–8]	2.0 ± 0.6 2.0 [0–3]	5.0 ± 0.8 5.0 [3–7]	1.6 ± 0.6 2.0 [0–2]	5.0 ± 0.3 5.0 [4–6]	5.8 ± 0.4 6.0 [5–6]	6.9 ± 0.6 7.0 [5–8]
Temporary	3.2 ± 0.5 3.0 [2–4]	3.1 ± 0.3 3.0 [3–4]	1.5 ± 0.6 2.0 [0–2]	3.6 ± 1.0 3.5 [2–5]	7.4 ± 1.1 7.0 [5–9]	6.0 ± 0.8 6.0 [4–7]	2.2 ± 0.7 2.0 [1–3]	5.0 ± 1.0 5.0 [3–6]	1.5 ± 0.6 2.0 [0–2]	5.0 ± 0.4 5.0 [4–6]	5.6 ± 0.6 6.0 [4–6]	6.9 ± 0.7 7.0 [5–8]
*p*	0.465	0.538	0.909	0.028	0.366	0.946	0.137	0.360	0.668	0.215	0.059	0.910

**Notes.**

VAS scores range from 0 (not affected at all) to 10 (severely affected), with higher scores indicating greater difficulty in performing activities of daily living.

## Discussion

The study revealed that patients with colostomies experienced varying degrees of difficulty in their daily living activities. According to the findings, it was determined that patients experienced the most difficulty particularly in areas such as excretion, anxieties about death and the future—maintaining quality of life, and spiritual needs (Table 2). This finding is consistent with studies in the literature and shows that individuals with stomas experience serious difficulties in the excretion process due to factors such as fear of leakage, concern about bad odor, and stoma bag management (Indrebøet al., 2023; Jeppesen et al., 2022; Down et al., 2021). In addition, it is reported that they experience anxiety, future concerns, a decrease in quality of life, and inadequate spiritual support at the psychosocial level, and that the needs in this regard are high (Osborne et al., 2022; Cengiz & Bahar, 2017; Brady et al., 2024).

The activities with the least difficulty were determined to be respiration, maintaining body temperature, and work-leisure. The literature also shows that these activities are less affected because they are not directly related to the stoma (Mena-Jiménez, Rodríguez-Suárez & González-de la Torre, 2024). However, moderate difficulty was observed in providing and maintaining a safe environment, communication, eating and drinking, personal hygiene, movement, sexuality, and sleep-rest.

Difficulties in personal hygiene were associated with body image changes and stoma-related physical limitations (Kalayci & Duruk, 2022; Sales et al., 2014). It is thought that sleep-rest problems may stem from nighttime concerns about the stoma bag and reduced comfort levels (Avci Işik et al., 2023; Temiz et al., 2022).

In the study, it was determined that activities related to sexuality were moderately affected for all patients. Changes in body image, reduced self-confidence, and relationship challenges with partners negatively affected sexual life (Petersén & Carlsson, 2021; Quemba Mesa et al., 2022; Lin, Yin & Chen, 2023). Similarly, Lin, Yin & Chen (2023) revealed that sexual life after stoma is significantly affected due to sexual problems caused by changes in physical function and psychological disorders, changes in the relationship with the partner, and the need for sexual life cognition and sexual knowledge. These findings highlight the importance of counseling on sexuality for individuals with stomas (Quemba Mesa et al., 2022).

No significant differences were found between gender, age, presence of chronic disease, need for care, independence level, or use of assistive devices in daily activity scores. This finding indicates that individuals with stomas may encounter similar difficulties in their daily living activities regardless of demographic characteristics such as age and gender, or health conditions such as chronic disease and level of independence, as stated by Petersén & Carlsson (2021). However, the fact that patients with a higher level of education have a lower difficulty score in activities related to sexual life suggests the positive effect of education level on individuals’ perception of body image and adaptation processes (Lin et al., 2024). Similarly, it is reported in the literature that individuals with a high level of education have better adaptation to sexual life due to their easier access to health information (Quemba Mesa et al., 2022; Lin et al., 2024). The fact that single patients have lower difficulty scores in providing and maintaining a safe environment and in work-leisure activities compared to married patients suggests the influence of social support mechanisms. In the literature, it is stated that social support provided by individuals’ family and environment plays an important role in disease and health-related adaptation processes (Jin et al., 2024; Muhammad, Akpor & Akpor, 2022; Baykara, Demir & Karadag, 2020). The possibility that single individuals receive more support, especially from their mothers, fathers, and siblings, may support their easier adaptation to daily living activities. In contrast, the increased responsibilities of married individuals towards their spouses and children may lead them to prioritize their individual needs, causing them to experience more difficulty (Baykara, Demir & Karadag, 2020). This finding shows that married individuals may need additional psychosocial support mechanisms in the process of adapting to daily living activities (Brady et al., 2024; Jin et al., 2024). In addition, the fact that patients with temporary stomas have lower difficulty scores in eating and drinking activities can be explained by the fact that individuals with temporary stomas experience fewer adaptation problems compared to those with permanent stomas. This finding parallels studies that address the psychological differences between temporary and permanent stomas (Simpson et al., 2023; Cengiz & Bahar, 2017; Choi et al., 2023).

This study contributes to the existing literature by providing a comprehensive assessment of daily living activity difficulties experienced by individuals with colostomy through a holistic nursing model (Roper-Logan-Tierney). Unlike previous studies focusing only on specific domains such as physical or psychological problems, this study integrates physical, psychosocial, and spiritual dimensions, offering a multidimensional understanding of patients’ adaptation processes.

## Conclusion

In conclusion, this study revealed that individuals with colostomy experience significant difficulties in daily living activities, particularly in excretion, anxiety about death and the future, and spiritual needs. In addition, it was observed that basic life activities such as personal hygiene, sleep-rest, and sexuality were also affected. The findings highlight the necessity of a holistic approach addressing physical, psychosocial, and spiritual aspects in post-colostomy care.

## Strengths and Limitations of the Study

In this study, various measures were taken to minimize methodological biases. Participants were given detailed information about the purpose, method, and confidentiality principles of the study, and it was emphasized that their responses would be kept anonymous. To reduce non-response bias, participants who could not be reached were called back at different times. To prevent social desirability bias, participants were informed that there were no right or wrong answers and that the study was only aimed at determining the difficulties in daily living activities. In addition, expressions that could trigger social desirability were avoided in the questionnaire form to reduce the tendency to give positive responses to healthcare professionals. In the data collection process, questions were ensured to be understood through face-to-face interviews in the first stage, and the risk of systematic error was reduced with telephone interviews in the second stage.

However, the study has some limitations. The research was conducted with a specific patient group, and the generalizability of the findings is limited. In addition, reliance on participants’ subjective statements may increase the risk of bias. Because the study employed a cross-sectional design, causal relationships between variables could not be established. Additionally, as some of the patient-reported outcome measures (PROMs), particularly the adapted DLA–VAS form, were used for the first time in this specific population, further validation studies are warranted. In future research, evaluations with larger and more diverse samples can increase the validity of the findings.

## Implications for Practice and Further Research

This study provides an important step in understanding daily living difficulties among individuals with colostomy and offers practical insights for healthcare professionals. Recognizing the influence of demographic and social support factors can guide individualized post-colostomy care strategies. Nurses and healthcare teams could design interventions that improve patients’ participation and adaptation in daily activities.

Future studies should test these findings in diverse populations and cultural settings. In-depth analysis of psychosocial and behavioral factors influencing daily living can contribute to the development of targeted nursing interventions. Large-scale longitudinal studies may also help clarify the long-term effects of social support and education level on recovery and adaptation after colostomy.

##  Supplemental Information

10.7717/peerj.20763/supp-1 Supplemental Information 1 STROBE Reporting Guidelines

10.7717/peerj.20763/supp-2 Supplemental Information 2 Colostomy Patients’ Daily Living Activity Problems and VAS Scores Dataset This dataset contains Visual Analog Scale (VAS) scores and related variables assessing difficulties in daily living activities among individuals with colostomy. The file includes demographic information, specific activity-related problem scores, and overall VAS ratings. Data were collected to evaluate the frequency and severity of challenges experienced in daily routines. The dataset is anonymized and intended for research and statistical analysis.

10.7717/peerj.20763/supp-3 Supplemental Information 3 Daily Living Activities Difficulty Level Assessment Form (ADL-VAS)

## Additional Information and Declarations

### Data Availability

The following information was supplied regarding data availability: The colostomy data is available in the Supplemental File and at Zenodo: Gülşen, M., Nursevim, A., Sevban, A., & Hülya, B. (2025). Evaluation of problems encountered in daily living activities by ındividuals with colostomy: use of the visual analog scale (1.0) [Data set]. Zenodo. https://doi.org/10.5281/zenodo.17634897
gülşen, . muaz . (2025). Evaluation of Problems Encountered in Daily Living Activities by Individuals with Colostomy: Use of the Visual Analog Scale [Data set]. Zenodo. https://doi.org/10.5281/zenodo.15800220.

## References

[ref-1] Abdul Khadar TF, Ramalingam V (2024). Effectiveness of the specific mobility exercises on pain intensity and quality of life among stoma patients: a quasi-experimental study. Cureus.

[ref-2] Aboma D, Kaba M (2023). Colostomy patient lived experience at public hospitals of Addis Ababa. Ethiopia: Phenomenology. Open Access Surgery.

[ref-3] Akel BS, Öksüz Ç, Karahan S, Düger T, Kayihan H (2012). Reliability and validity of milliken activities of daily living scale (MAS) in measuring activity limitations of a turkish population. Scandinavian Journal of Occupational Therapy.

[ref-4] Albersnagel FA (1998). Velten and musical mood induction procedures: a comparison with accessibility of thought associations. Behaviour Research and Therapy.

[ref-5] Alp R (2014). Evaluation of the life model: the problems that stoma patients faced at home. Master’s thesis.

[ref-6] Australian Association of Stomal Therapy Nurses-AASTN (2025). Clinical guidelines for stomal therapy nursing practice. https://stomaltherapy.au/wp-content/uploads/2022/03/2013-Clinical-Guidelines-Book.pdf.

[ref-7] Avci Işik S, Balanuye B, Budak Ertürk E, Karahan A, Üstündağ Ç, Uğurlu Z, Koç MA (2023). Sleep problems in individuals with intestinal stomas and determining the quality of sleep: a multicenter study. Journal of Wound, Ostomy, and Continence Nursing.

[ref-8] Aydın A, Araz A, Aslan A (2011). Visual analog scale and emotion lattice: adaptation to our culture. Turkish Psychology Writings.

[ref-9] Babaoğlu AB, Tekindal M, Büyükuysal MÇ, Tözün M, Elmalı F, Bayraktaroğlu T, Tekindal MA (2021). Epidemiyolojide Gözlemsel Çalışmaların Raporlanması: STROBE Kriterlerinin Türkçe Uyarlaması. Medical Journal of Western Black Sea.

[ref-10] Baykara ZG, Demir S, Karadag A (2020). Family functioning, perceived social support, and adaptation to a stoma: a descriptive, cross-sectional survey. Wound Management & Prevention.

[ref-11] Begum MR, Hossain MA (2019). Validity and reliability of visual analogue scale (VAS) for pain measurement. Journal of Medical Case Reports and Reviews.

[ref-12] Brady RRW, Sheard D, Alty M, Vestergaard M, Boisen EB, Ainsworth R, Hansen HD, Ajslev TA (2024). Evaluating the effect of a novel digital ostomy device on leakage incidents, quality of life, mental well-being, and patient self-care: an interventional, multicentre clinical trial. Journal of Clinical Medicine.

[ref-13] Cengiz B, Bahar Z (2017). Perceived barriers and home care needs when adapting to a fecal ostomy: a phenomenological study. Journal of Wound, Ostomy, and Continence Nursing.

[ref-14] Choi HR, Park HS, Hong Y, Kim YA (2023). The lived experience of colorectal cancer patients with a temporary ileostomy and the patient’s perception of the ostomy nurses’ educational interventions. Supportive Care in Cancer.

[ref-15] Colostomy UK (2019). Living with a stoma. https://www.colostomyuk.org/wp-content/uploads/2025/12/Living-with-a-stoma.pdf.

[ref-16] Down G, Vestergaard M, Ajslev TA, Boisen EB, Nielsen LF (2021). Perception of leakage: data from the Ostomy Life Study 2019. British Journal of Nursing (Mark Allen Publishing).

[ref-17] Duluklu B, Çelik SŞ (2024). Lived experiences of patients after colorectal cancer and permanent colostomy: a parallel-design mixed-methods study. Advances in Skin & Wound Care.

[ref-18] Erdoğan S, Nahcıvan N, Esin N (2014). Hemşirelikte Araştırma Süreci, Uygulama ve Kritik.

[ref-19] Fairbank JC, Couper J, Davies JB, O’Brien JP (1980). The Oswestry low back pain disability questionnaire. Physiotherapy.

[ref-20] Gélinas I, Gauthier L, McIntyre M, Gauthier S (1999). Development of a functional measure for persons with Alzheimer’s disease: the disability assessment for dementia. American Journal of Occupational Therapy.

[ref-21] Gülşen M, Akansel N (2020). Effects of discharge education and telephone follow-up on cataract Patients’ activities according to the model of living. Journal of Perianesthesia Nursing.

[ref-22] Hamad B (2025). Lived experiences of iraqi patients with permanent colostomy: a phenomenological study: lived experiences of iraqi patients with permanent colostomy. Journal of Contemporary Medical Sciences.

[ref-23] Indrebø KL, Aasprang A, Olsen TE, Andersen JR (2023). Factors associated with leakage in patients with an ostomy: a cross-sectional study. Nursing Open.

[ref-24] Jeppesen PB, Vestergaard M, Boisen EB, Ajslev TA (2022). Impact of stoma leakage in everyday life: data from the Ostomy Life Study 2019. British Journal of Nursing (Mark Allen Publishing).

[ref-25] Jin Y, Li X, Ma H, Xiong L, Zhao M, Wang H (2024). Dyadic effects of perceived stress, relationship satisfaction and distress disclosure on emotional distress in colorectal cancer patients and their family caregivers: an actor-partner interdependence mediation model. Asia-Pacific Journal of Oncology Nursing.

[ref-26] Kalayci F, Duruk N (2022). Assessment of the difficulties experienced by individuals with intestinal stomas: a qualitative study. Advances in Skin & Wound Care.

[ref-27] Kara B, Aslan FE (2017). Investigation of the stoma individuals home first day experience. Turkish Journal of Colorectal Disease.

[ref-28] Katz S, Ford AB, Moskowitz RW, Jackson BA, Jaffe MW (1963). Studies of illness in the aged. The index of ADL: a standardised measure of biological and psychosocial function. Journal of the American Medical Association.

[ref-29] Lawton MP, Brody EM (1969). Assessment of older people: self-maintaining and instrumental activities of daily living. The Gerontologist.

[ref-30] Lin L, Fang Y, Wei Y, Huang F, Zheng J, Xiao H (2024). The effects of a nurse-led discharge planning on the health outcomes of colorectal cancer patients with stomas: a randomized controlled trial. International Journal of Nursing Studies.

[ref-31] Lin S, Yin G, Chen L (2023). The sexuality experience of stoma patients: a meta-ethnography of qualitative research. BMC Health Services Research.

[ref-32] Mena-Jiménez AV, Rodríguez-Suárez CA, González-de la Torre H (2024). Return to physical activity in individuals with surgical stomas: a scoping review. Sports.

[ref-33] Muhammad FA, Akpor OA, Akpor OB (2022). Lived experiences of patients with ostomies in a University Teaching Hospital in Kwara State, Nigeria. Heliyon.

[ref-34] Nurses Specialized in Wound, Ostomy and Continence Canada-NSWOCC (2022). A guide to living with a colostomy (2nd ed.).

[ref-35] Osborne W, White M, Aibibula M, Boisen EB, Ainsworth R, Vestergaard M (2022). Prevalence of leakage and its negative impact on quality of life in people living with a stoma in the UK. British Journal of Nursing (Mark Allen Publishing).

[ref-36] Petersén C, Carlsson E (2021). Life with a stoma-coping with daily life: experiences from focus group interviews. Journal of Clinical Nursing.

[ref-37] Quemba Mesa MP, Diaz Fernández JK, Vargas Rodríguez LY, Bautista Plazas L, Pulido Barragán SP (2022). Experiences and perceptions in dyads about ostomy care. Meta-synthesis of qualitative studies. Investigacion Y Educacion En Enfermeria.

[ref-38] Sales PM, Carvalho AF, McIntyre RS, Pavlidis N, Hyphantis TN (2014). Psychosocial predictors of health outcomes in colorectal cancer: a comprehensive review. Cancer Treatment Reviews.

[ref-39] Sant’Anna T, Donária L, Furlanetto KC, Morakami F, Rodrigues A, Grosskreutz T (2017). Development, validity and reliability of the londrina activities of daily living protocol for subjects with COPD. Respiratory Care.

[ref-40] Simpson E, Pourshahidi K, Davis J, Slevin M, Lawther R, O’Connor G, Porrett T, Marley J, Gill C (2023). Living with and without an intestinal stoma: factors that promote psychological well-being and self-care: a cross-sectional study. Nursing Open.

[ref-41] Soelling SJ, Rubio-Chavez A, Ingram Z, Baird L, Brindle ME, Cooper Z, Vranceanu AM, Ritchie CS, Cauley CE (2025). Challenges faced by patients undergoing fecal ostomy surgery: a qualitative study of colorectal cancer patient perspectives. Journal of Gastrointestinal Surgery.

[ref-42] Stavropoulou A, Vlamakis D, Kaba E, Kalemikerakis I, Polikandrioti M, Fasoi G, Vasilopoulos G, Kelesi M (2021). “Living with a stoma”: exploring the lived experience of patients with permanent colostomy. International Journal of Environmental Research and Public Health.

[ref-43] Temiz Z, Cavdar I, Ozbas A, Altunsoy M, Akyuz N, Kutlu FY (2022). Sleep quality and factors affecting sleep in individuals with an intestinal ostomy: a descriptive cross-sectional study. Wound Management & Prevention.

[ref-44] The jamovi project (2024). https://www.jamovi.org.

[ref-45] Tufekci H, Akansel N, Sivrikaya SK (2022). Pain interference with daily living activities and dependency level of patients undergoing CABG surgery. Pain Management Nursing.

[ref-46] United Ostomy Associations of America (UOAA) (2024). New ostomy patient guide. https://www.ostomy.org/wp-content/uploads/2024/04/UOAA-New-Ostomy-Patient-Guide-2024-04.pdf.

[ref-47] Wang SM, Jiang JL, Li R, Wang JJ, Gu CH, Zeng J, Wei XH, Chen M (2024). Qualitative exploration of home life experiences and care needs among elderly patients with temporary intestinal stomas. World Journal of Gastroenterology.

[ref-48] World Council of Enterostomal Therapists-WCET (2025). International Ostomy Guidelines, 2020. https://wcetn.org/page/InternationalOstomyGuidelines.

[ref-49] Yang F, Cui S, Cai M, Feng F, Zhao M, Sun M, Zhang W (2024). The experiences of family resilience in patients with permanent colostomy and their spouses: a dyadic qualitative study. European Journal of Oncology Nursing.

[ref-50] Zhabagin K, Zhabagina A, Shalgumbayeva G, Toleutayeva D, Baissalbayeva A, Toleutayev T, Telmanova Z, Igissin N, Moore M (2024). Quality of life of colorectal cancer patients: a literary review. Iranian Journal of Public Health.

