# Supplementary material for: Evaluation of problems encountered in daily living activities by ındividuals with colostomy: use of the Visual Analog Scale
Source: PeerJ. 2026 Feb 11;14:e20763. doi: 10.7717/peerj.20763 (PMC12906260; doi:10.7717/peerj.20763)
Supplement: Supplemental Information 3 [file peerj-14-20763-s003.docx]

| We would like to clarify that the “Daily Living Activities Difficulty Assessment Form (GYA-VAS)” is an **original tool developed specifically for this study** by the research team. It is not an adapted or translated version of a copyrighted scale, and therefore **no permission from a copyright holder is required**.  The development of this form was theoretically based on **Roper, Logan, and Tierney’s model of living**, which defines activities of daily living as key components of patient care. The tool utilizes a **Visual Analog Scale (VAS)** format, a commonly used and **public domain measurement technique**, to assess the perceived difficulty in performing daily activities among individuals with a colostomy.  Given that both the conceptual framework (Roper et al.) and the measurement approach (VAS) are **well-established and not copyright-protected**, we confirm that no specific license or permission is necessary. However, we have clearly acknowledged the theoretical foundation in the manuscript and would be happy to provide any further clarification upon request. | | | | | | | | | | | | | | | | |
| --- | --- | --- | --- | --- | --- | --- | --- | --- | --- | --- | --- | --- | --- | --- | --- | --- |
| **Post-Discharge Activities of Daily Living Questionnaire Form** | **EVALUATION** | | | | | | | | | | | | | | | |
| **1. Problems and difficulties in providing and maintaining a safe environment** | 0 1 2 3 4 5 6 7 8 9 10  0 points: I had no trouble ---------- 10 points: I had extreme difficulty | | | | | | | | | | | | | | | **Center** |
| Level of difficulty in providing pain management |  |  |  |  |  |  |  |  |  |  |  |  |  |  |  |  |
| Level of difficulty in medication management |  |  |  |  |  |  |  |  |  |  |  |  |  |  |  |  |
| Level of difficulty in controlling bleeding |  |  |  |  |  |  |  |  |  |  |  |  |  |  |  |  |
| Level of difficulty in maintaining skin integrity around the stoma |  |  |  |  |  |  |  |  |  |  |  |  |  |  |  |  |
| Level of difficulty in procurement of stoma care materials |  |  |  |  |  |  |  |  |  |  |  |  |  |  |  |  |
| Level of difficulty in infection management |  |  |  |  |  |  |  |  |  |  |  |  |  |  |  |  |
| Level of difficulty in managing emergencies |  |  |  |  |  |  |  |  |  |  |  |  |  |  |  |  |
| **2. Problems in communication-related activities and level of difficulty** | 0 1 2 3 4 5 6 7 8 9 10  0 points: I had no trouble ---------- 10 points: I had extreme difficulty | | | | | | | | | | | | | | | **Center** |
| Level of difficulty in verbal communication after surgery |  |  |  |  |  |  |  |  |  |  |  |  |  |  |  |  |
| Level of difficulty in phone calls |  |  |  |  |  |  |  |  |  |  |  |  |  |  |  |  |
| Level of difficulty in expressing oneself |  |  |  |  |  |  |  |  |  |  |  |  |  |  |  |  |
| **3. Problems in respiratory-related activities and level of difficulty** | 0 1 2 3 4 5 6 7 8 9 10  0 points: I had no trouble ---------- 10 points: I had extreme difficulty | | | | | | | | | | | | | | | **Ort** |
| Level of difficulty during inhalation and exhalation |  |  |  |  |  |  |  |  |  |  |  |  |  |  |  |  |
| Level of difficulty during coughing, sneezing and deep breathing |  |  |  |  |  |  |  |  |  |  |  |  |  |  |  |  |
| **4. Problems in activities related to eating and drinking and level of difficulty** | 0 1 2 3 4 5 6 7 8 9 10  0 points: I had no trouble ---------- 10 points: I had extreme difficulty | | | | | | | | | | | | | | | **Center** |
| Level of difficulty in nutrition management |  |  |  |  |  |  |  |  |  |  |  |  |  |  |  |  |
| Level of difficulty in fluid management |  |  |  |  |  |  |  |  |  |  |  |  |  |  |  |  |
| Level of difficulty in vitamin and mineral intake |  |  |  |  |  |  |  |  |  |  |  |  |  |  |  |  |
| **5. Problems in activities related to excretion and level of difficulty** | 0 1 2 3 4 5 6 7 8 9 10  0 points: I had no trouble ---------- 10 points: I had extreme difficulty | | | | | | | | | | | | | | | **Center** |
| Level of difficulty in managing diarrhea and constipation |  |  |  |  |  |  |  |  |  |  |  |  |  |  |  |  |
| Level of difficulty in maintaining gas control |  |  |  |  |  |  |  |  |  |  |  |  |  |  |  |  |
| Level of strain from the stoma emptying process |  |  |  |  |  |  |  |  |  |  |  |  |  |  |  |  |
| **6. Problems in individual hygiene-related activities and level of difficulty** | 0 1 2 3 4 5 6 7 8 9 10  0 points: I had no trouble ---------- 10 points: I had extreme difficulty | | | | | | | | | | | | | | | **Center** |
| Level of difficulty during dressing and undressing |  |  |  |  |  |  |  |  |  |  |  |  |  |  |  |  |
| Level of difficulty in bathing |  |  |  |  |  |  |  |  |  |  |  |  |  |  |  |  |
| Level of difficulty in maintaining stoma hygiene |  |  |  |  |  |  |  |  |  |  |  |  |  |  |  |  |
| **7. Having problems with body temperature and level of difficulty** | 0 1 2 3 4 5 6 7 8 9 10  0 points: I had no trouble ---------- 10 points: I had extreme difficulty | | | | | | | | | | | | | | | **Center** |
| Level of difficulty in choosing clothes |  |  |  |  |  |  |  |  |  |  |  |  |  |  |  |  |
| Level of difficulty in managing temperature change during skin contact with the stoma bag |  |  |  |  |  |  |  |  |  |  |  |  |  |  |  |  |
| **8. Problems in movement-related activities and level of difficulty** | 0 1 2 3 4 5 6 7 8 9 10  0 points: I had no trouble ---------- 10 points: I had extreme difficulty | | | | | | | | | | | | | | | **Center** |
| Level of difficulty going up and down stairs |  |  |  |  |  |  |  |  |  |  |  |  |  |  |  |  |
| Level of difficulty in walking-exercise |  |  |  |  |  |  |  |  |  |  |  |  |  |  |  |  |
| Level of difficulty in driving |  |  |  |  |  |  |  |  |  |  |  |  |  |  |  |  |
| **9. Problems and level of difficulty in activities related to work and leisure** | 0 1 2 3 4 5 6 7 8 9 10  0 points: I had no trouble ---------- 10 points: I had extreme difficulty | | | | | | | | | | | | | | | **Center** |
| Level of difficulty in activities such as watching TV and reading books |  |  |  |  |  |  |  |  |  |  |  |  |  |  |  |  |
| Level of difficulty in social activities (cinema, theater, concerts, hiking) |  |  |  |  |  |  |  |  |  |  |  |  |  |  |  |  |
| Level of difficulty in housework, gardening and all activities in professional life |  |  |  |  |  |  |  |  |  |  |  |  |  |  |  |  |
| **10. Problems in sexuality-related activities and level of difficulty** | 0 1 2 3 4 5 6 7 8 9 10  0 points: I had no trouble ---------- 10 points: I had extreme difficulty | | | | | | | | | | | | | | | **Center** |
| Level of difficulty in adapting to sexual activity |  |  |  |  |  |  |  |  |  |  |  |  |  |  |  |  |
| Level of difficulty in emotional and physical intimacy with partner |  |  |  |  |  |  |  |  |  |  |  |  |  |  |  |  |
| Level of difficulty in adapting to the change in body perception due to stoma |  |  |  |  |  |  |  |  |  |  |  |  |  |  |  |  |
| Experiencing pregnancy and level of difficulty in managing it |  |  |  |  |  |  |  |  |  |  |  |  |  |  |  |  |
| **11. Problems in activities related to sleep and rest and level of difficulty** | 0 1 2 3 4 5 6 7 8 9 10  0 points: I had no trouble ---------- 10 points: I had extreme difficulty | | | | | | | | | | | | | | | **Center** |
| Difficulty falling asleep and resting |  |  |  |  |  |  |  |  |  |  |  |  |  |  |  |  |
| Level of difficulty in adjusting sleeping and resting position |  |  |  |  |  |  |  |  |  |  |  |  |  |  |  |  |
| **12. Concerns about death and having problems with spiritual needs and level of difficulty** | 0 1 2 3 4 5 6 7 8 9 10  0 points: I had no trouble ---------- 10 points: I had extreme difficulty | | | | | | | | | | | | | | | **Center** |
| Level of difficulty in managing future anxiety |  |  |  |  |  |  |  |  |  |  |  |  |  |  |  |  |
| Level of difficulty in managing quality of life |  |  |  |  |  |  |  |  |  |  |  |  |  |  |  |  |
| Level of difficulty in worshipping |  |  |  |  |  |  |  |  |  |  |  |  |  |  |  |  |
